# Shifting social norms to prevent age-disparate transactional sex in Tanzania: what we can learn from intervention development research

**DOI:** 10.3389/fpsyg.2023.926531

**Published:** 2023-05-02

**Authors:** Lottie Howard-Merrill, Cathy Zimmerman, Revocatus Sono, John Riber, Joyce Wamoyi, Piotr Pawlak, Lori Rolleri Insignares, Robyn Yaker, Ana Maria Buller

**Affiliations:** ^1^Department of Global Health and Development, Faculty of Public Health and Policy, London School of Hygiene and Tropical Medicine, London, United Kingdom; ^2^Department of Education, Practice and Society, Institute of Education, University College London, London, United Kingdom; ^3^Amani Girls Home, Mwanza, Tanzania; ^4^Media for Development International, Arusha, Tanzania; ^5^Department for Sexual and Reproductive Health, National Institute of Medical Research, Dar es Salaam, Tanzania; ^6^Independent Consultant, Washington, DC, United States; ^7^Lori Rolleri Consulting Inc., New York City, GA, United States; ^8^HIAS, New York, NY, United States

**Keywords:** social norms, age-disparate transactional sex, sexual exploitation, adolescence, reproductive and sexual health, intervention development, mass media interventions, curriculum-based intervention

## Abstract

This paper reflects on the development process (2015–2020) of the Learning Initiative for Norms, Exploitation, and Abuse (LINEA) Intervention. The LINEA Intervention is a multi-component social norms intervention to prevent age-disparate transactional sex in Tanzania. This paper aims to: (1) critically reflect on the LINEA Intervention development process by retrospectively comparing it with a pragmatic, phased framework for intervention development in public health, the Six Essential Steps for Quality Intervention Development (6SQuID); and (2) discuss the usefulness and applicability of this framework to guide intervention development for gender-based violence prevention. This paper contributes to a growing field of intervention development research to improve the designs of interventions to prevent gender-based violence. Findings showed that the LINEA Intervention development approach mostly aligned with the steps in 6SQuID framework. However, the LINEA Intervention development process placed particular emphasis on two phases of the 6SQuID framework. First, the LINEA Intervention development process included significant investment in formative research, feasibility testing, and refinement; and second, the LINEA Intervention was informed by a clearly articulated behavior change theory—social norms theory. Beyond the 6SQuID framework the LINEA Intervention development process: (i) followed a non-linear, iterative process; (ii) applied ongoing feasibility testing to refine the intervention, and (iii) relied on co-development with local implementers and participants. This paper suggests future components for a robust intervention development process, highlighting beneficial additions to the 6SQuID approach, a well-recognized intervention development sequence. Particularly useful additions include incorporating sufficient time, flexibility, and resources to foster meaningful collaborations and iteration on the intervention design.

## 1. Introduction

Evidence on interventions to prevent gender-based violence (GBV) in low-and middle-income countries has increased substantially over the past decade with growing consensus about what works in different settings (Heise, 2011; Jewkes et al., 2021). However, it is not uncommon for GBV interventions, and public health interventions more generally, to be implemented or adapted to new settings and subjected to evaluation before there is sufficient evidence that they are well-targeted, address modifiable determinants, and meet the needs of intervention participants. Furthermore, interventions have been critiqued for overlooking the influence of contextual factors and possible unintended consequences (Pawson and Tilley, 1997; Zimmerman et al., 2021). A growing number of intervention evaluations have exposed the need for stronger intervention development research to ensure interventions can be well-targeted, with optimal efficacy and potential for replication and scale-up (Onken et al., 2014; Zimmerman et al., 2016; Bleijenberg et al., 2018; Turner et al., 2019; Zimmerman et al., 2021). Hoddinott (2015) defines intervention development research as:

*A study that describes the rationale, decision-making processes, methods, and findings which occur between the idea or inception of an intervention until it is ready for formal feasibility, pilot, or efficacy testing prior to a full trial or evaluation. (2015, p.1)*

In recent years, researchers have started to publish approaches to intervention development. In a systematic review O’Cathain et al. (2019) synthesized this literature to identify eight categories of approach to intervention development. Although many of the approaches they identified could be applied to the LINEA intervention we have chosen to reflect on a stepped or phased approach, as a systematic, practical, logical, and evidence-based guide to intervention development. Phased models for intervention development are thought to maximize effectiveness and reduce waste for intervention implementation and evaluation (Onken et al., 2014; Wight et al., 2016; Bleijenberg et al., 2018; O’Cathain et al., 2019; Skivington et al., 2021). Phased models also describe each stage’s relative importance and clarify language to facilitate mutual understanding between researchers and implementers (Onken et al., 2014).

The framework described by Wight et al. (2016) Six Essential Steps for Quality Intervention Development (6SQuID) has been recognized as a pragmatic guide for the development of complex interventions in public health. The 6SQuID framework describes an intervention development process from the inception of the idea to preparation of the prototype for implementation and evaluation. The key components of the 6SQuID framework are summarized in Table 1.

**Table 1:** Six Essential Steps for Quality Intervention Development (6SQuID) stages in intervention development, adapted from Wight et al. (2016).

6SQuID framework stages in intervention development	
1	**Define and understand the problem and its causes.** Identify ways to define and measure ‘the problem’, including establishing whether the focus is the health risk factor or health outcome. Assess the problem’s causes and distribution within a community in consultation with key stakeholders.
2	**Identify how the intervention will interact with the system.** Establish causal or contextual factors at the individual, inter-personal institutional, and structural levels. Consider both immediate and underlying factors that shape a problem. Decide which factors are modifiable and are most likely to influence change. Identify which target population will respond best to the intervention.
3	**Decide on change mechanisms.** Decide on and clearly articulate change mechanisms for the modifiable factors chosen in Stage 2, by depicting the program theory in a theory of change. Program theories should be informed by formalized theories of behavior change with predictive and explanatory power.
4	**Clarify how to deliver change mechanisms.** Work out how to deliver the change mechanisms and develop an implementation plan with stakeholders. Clarify the conditions and resources necessary for successful implementation in conjunction with stakeholders. Anticipate and minimize any harmful unintended consequences.
5	**Test and refine the intervention on a small scale.** Establish acceptability for the intervention among the intervention participants, practitioners, and implementing organizations. Finalize the intervention components and duration. Conduct testing and adaptations incrementally.
6	**Collect evidence of effectiveness to justify implementation and evaluation.** Consider whether small-scale implementation of the intervention is working as intended and achieving some short-term outcomes. Monitor and respond to any negative potential unintended consequences, before implementing and evaluating on a larger scale.

In this paper, we use the 6SQuID framework to retrospectively reflect on the intervention development process (2015–2020) for the Learning Initiative on Norms, Exploitation and Abuse (LINEA) Intervention in North-western Tanzania. The LINEA Intervention aims to prevent age-disparate transactional sex (ADTS) using a social norms approach. The LINEA Intervention is one component of the broader LINEA initiative, which has an expressed aim of testing the application of social norms theory to prevent sexual exploitation of children and adolescents.

We are applying the 6SQuID framework retrospectively primarily because the framework had not been published at the inception of the LINEA initiative (2013–14). During the LINEA intervention development process the field of intervention development research has moved on significantly, and the value of reviewing the literature on approaches to intervention development has gained traction only recently. The LINEA Intervention development approach emerged organically drawing on learning from existing and successful interventions to prevent gender-based violence, including SASA, IMAGE and MAISHA (Pronyk et al., 2006; Abramsky et al., 2014; Harvey et al., 2021), our review of the literature on social norms change theory, and the existing evidence about the drivers of ADTS.

### 1.1. Learning Initiative on Norms Exploitation and Abuse: background and rationale

The Learning Initiative on Norms, Exploitation, and Abuse (LINEA) Intervention drew on social norms theories to promote behavior change (Cislaghi and Heise, 2018; Glass et al., 2019; Clark et al., 2020). Social norms are defined as reciprocally held beliefs and expectations about what others do (*descriptive norms*) and what others should do (*injunctive norms*), (Cialdini and Trost, 1998; Mackie et al., 2015; Horne and Mollborn, 2020). Social norms are maintained through multiple mechanisms that reflect entrenched power hierarchies (e.g., patriarchal power inequalities), (Harper et al., 2020). Social norms operate within *reference groups*, defined as the group of people an individual thinks set and maintain the expectations related to a given social norm. Social norms are enforced within reference groups through the anticipation of *sanctions:* those who adhere to norms are rewarded, and those who do not are punished (Bicchieri, 2005; Horne and Mollborn, 2020). Intervening at the multiple levels where social norms operate (individual, social and institutional) offers promising opportunities for change (Cislaghi and Heise, 2018).

The LINEA Intervention was designed to explore the application of social norms theory to prevent the sexual exploitation of children and adolescents. Following a global systematic review of the social norms linked to the sexual exploitation of children and adolescents (Buller et al., 2020), the LINEA Intervention was designed to focus on one target behavior, age-disparate transactional sex (ADTS). Transactional sex is defined as non-commercial sexual relationships or sex acts outside of marriage based on the implicit understanding that material support or other benefits will be exchanged for sex (Stoebenau et al., 2016). We define ADTS as transactional sex occurring between adolescent girls under the age of 18 and adult men more than 10 years older. Negative consequences of ADTS for adolescent girls include: increased risk of HIV and sexually transmitted infections (STIs); unplanned pregnancy; abortion; child marriage; school dropout; social sanctions; intimate partner violence; and sexual coercion (Luke and Kurz, 2002; Tener, 2019; Muthoni et al., 2020; UNAIDS, 2020; Kyegombe et al., 2020a).

To date, there is limited evidence about what works to prevent ADTS in Sub-Saharan Africa (Kaufman et al., 2013; Pettifor et al., 2019; Muthoni et al., 2020). To our knowledge, there are no rigorously evaluated interventions that target ADTS prevention as the primary outcome, that use a social norms approach, or that work simultaneously with men and adolescent girls. The LINEA Intervention results contribute evidence to inform the future design of interventions to prevent ADTS (Turner et al., 2019).

### 1.2. The LINEA Intervention

**Figure 1:**
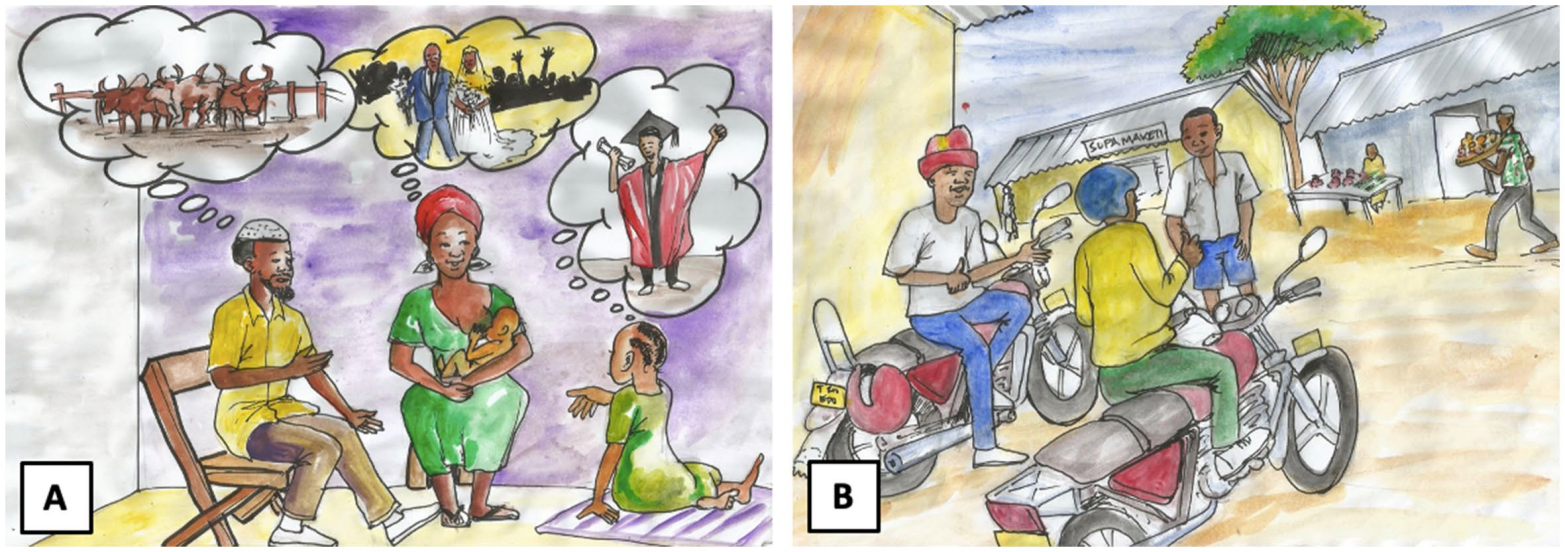
Storyboard images of learning initiative on norms, exploitation, and abuse (LINEA) radio drama characters: On the left, the main adolescent girl character, Amali, navigates the expectations of her family members **(A)**. On the right, the main adult man character, Tuma, must resist peer pressure to avoid age-disparate transactional sex (ADTS) **(B)**.

The LINEA Intervention is comprised of two components (Table 2). The first component is a 39-episode radio drama called *Msichana Wa Kati* (The Girl in the Middle), designed to shift social norms in the community. The second component includes two curricula designed to target individuals and small groups. Curricula content addresses determinants of ADTS, such as knowledge, skills, motivations, and social norms. Sessions cover topics such as gender equality and power, adolescent health and development, healthy relationships, communication, gender-based violence, transactional sex, and fatherhood and caregiving. Radio drama characters and storylines are incorporated throughout the curricula as case studies and examples and are also illustrated in curricula materials (see Figure 1 for examples). One curriculum engages adolescent girls (aged 13–15) in schools. The second curriculum works with adult men who are at least 10 years older than the youngest girls participating in the girls’ sessions (i.e., aged 23 and over). The male participants work in male-dominated activities and are the primary perpetrators and instigators of ADTS with girls. The curricula use interactive and participatory learning activities to build knowledge, skills, and support for new social norms. Examples include critical reflection discussions, role plays, skill practice, and small group work. The sessions in the curricula include take-home activities to facilitate the organized diffusion of learning and support for protective norms in the participants’ reference groups.

**Table 2:** Learning Initiative on Norms, Exploitation, and Abuse (LINEA) Intervention components.

	Component 1: community Level	Component 2: individual and small group level	
	Radio drama	Girls’ curriculum	Men’s curriculum
Target population	Whole community	Adolescent girls aged 13–15	Adult men 23 years old and over
Number of sessions/episodes	39-episodes	17-session curriculum	18-session curriculum
Length of sessions/episodes	20 min	90 min	120 min
Number of participants	Whole community	15–20	15–20
Duration	9 months	4 months	4 months

The LINEA Intervention was developed iteratively over multiple stages of testing and creation of intervention components. The collaborative approach to intervention design meant that LINEA worked with two locally-based implementing partners: Amani girls home (AGH) and Media for Development International, Tanzania (MFDI). We also gained input from researchers from the National Institute for Medical Research in Tanzania, curriculum development experts, and the community intended to participate in the finalized intervention.

## 2. Linea Intervention development research: key aims and methods

Data were collected to inform LINEA Intervention development research during three phases: (1) qualitative formative research; (2) feasibility testing; and (3) an iterative radio drama development process. Intervention materials, such as the curricula, were developed in parallel with research activities as explained in the following section. Data collection methods, data analysis, and ethical issues are also summarized in LINEA Evidence Brief 3 (LINEA, 2022).

The purpose of the first phase, qualitative formative research, was to understand the nature of the problem of ADTS in three geographical settings. We collected data to explore beliefs about whether ADTS is exploitative and social norms upholding the practice. The research was conducted in partnership with local research institutions in Tanzania, Uganda, and Brazil from 2016 to 17, and the methods and findings from the research in all three contexts have been reported elsewhere (Wamoyi et al., 2018, 2019; Howard-Merrill et al., 2020; Ignacio et al., 2020; Kyegombe et al., 2020a,b,c; Wamoyi et al., 2021; Perrin et al., 2022). Following this qualitative formative research phase we chose to conduct the LINEA Intervention development process in Tanzania.

The second phase, intervention feasibility testing, occurred in June–July 2019 in partnership with Amani Girls Home and aimed to test the LINEA Intervention proof of concept and explore intervention delivery. We also collected evidence on indications of change and unintended outcomes (LINEA, 2022). A pre-and post-research design was carried out (without a control group) in a peri-urban community in the Mwanza region of Tanzania.

Feasibility data about the curriculum were collected using three methods: (1) structured observations of curriculum sessions with one group comprising 15 adolescent girls and another with seven adult couples with an adolescent daughter; (2) interviews before and after curriculum participation to assess indications of change with three adolescent girls, three men, and three women participants; and (3) feedback from implementing partner staff. Data collected during feasibility testing informed the production of the final LINEA Intervention curricula.

Feasibility data about the radio drama were collected from a series of radio drama listener groups with four different populations: 14 adolescent girls, 10 women and nine men with adolescent daughters, and seven community leaders (two women and five men). During listener group sessions participants shared feedback on radio drama storylines, characters, and casting. The radio drama was finalized during the third stage of LINEA Intervention research in partnership with the radio drama production company MFDI, and implementing partner organization, AGH.

The third phase of intervention development was an iterative radio drama development process conducted in 2019 in the Mwanza region. This research was designed to develop and finalize the radio drama storylines (case study below). Feedback was collected during four listener group sessions with approximately 15 participants each: adolescent girls aged 13–15, adult women, adult men, and community leaders.

The LINEA Intervention development process is complete and at the time of writing pilot testing of the intervention is underway, including studies to explore delivery approaches transferability to different locations in Tanzania.

## 3. Applying the 6SQuID framework

In this section, we reflect on how LINEA Intervention development process compared to the 6SQuID framework stages.

### 3.1. Stage 1: defining and understanding the problem

The Learning Initiative on Norms, Exploitation and Abuse Intervention development commenced with a qualitative formative study with men and women, and adolescent boys and girls in the Mwanza region in 2016–17. The study explored local perspectives of ADTS. The formative research found that adolescent girls believed adult men would provide more and better gifts than same-age partners (Wamoyi et al., 2018, 2021). Adult men claimed to find adolescent girls more sexually attractive than their wives (Howard-Merrill et al., 2020). We also found that participation in ADTS boosts the status of some men and adolescent girls, while for others it is a source of shame and regret (Wamoyi et al., 2018, 2019; Howard-Merrill et al., 2020). Using this information, we identified the social norms underpinning ADTS (described in Stage 2).

The LINEA Intervention formative research aligned closely with Stage 1 of the 6SQuID framework: understanding the nature of the problem. Our findings indicated that transactional sex was considered exploitative if the girl was forced to have sex or if she was perceived to be particularly vulnerable and unable to meet her material needs (Wamoyi et al., 2019). We found that men were perceived to take advantage of their position of relative power and affluence to engage in ADTS (Wamoyi et al., 2019; Howard-Merrill et al., 2020). Our findings confirmed the current literature that suggests that while girls often display some agency related to partner choice, once they enter a transactional sex relationship, their power is significantly reduced (Jewkes and Morrell, 2012; Groes-Green, 2013; Ranganathan et al., 2018).

By learning how the community perceived the problem of ADTS and how these attitudes related to the academic literature, we went beyond what is suggested in 6SQuID framework, notably gaining insights from the LINEA data on men’s and boys’ motivations for and perceptions of ADTS. Comparing men’s motivations and perceptions with women’s accounts facilitated the intervention’s aim to target both adult men and adolescent girls.

### 3.2. Stage 2: identifying modifiable causal or contextual factors

Modifiable causal factors identified through the LINEA Intervention formative research were social norms linked to ADTS. We found that participation in ADTS in part represents men’s and girls’ adherence to social norms (Howard-Merrill et al., 2020). We identified four modifiable social norms that put girls at higher risk of ADTS: (1) girls are expected to obtain money, gifts, or other benefits from their sexual partner; (2) girls are expected to gain status through material items and other benefits accessed through ADTS; (3) girls who receive money, gifts, or other benefits from men are expected to reciprocate with sex; and (4) girls who have reached puberty are no longer children and therefore are perceived to be ready for sex (Wamoyi et al., 2019). We also identified two key social norms influencing ADTS among men: (1) men are expected to have heightened sexuality and sexual prowess; and (2) men are expected to provide economically in sexual relationships (Howard-Merrill et al., 2020).

When comparing this stage of the LINEA Intervention development process and 6SQuID, the 6SQuID framework suggests considering determinants of change at multiple levels (e.g., individual, inter-personal, and institutional-level changes). Likewise, the LINEA Intervention targets opportunities for change at multiple levels, including individual-level knowledge and skills required to act against ADTS and structural-level risk factors for ADTS. In alignment with the LINEA initiative’s central aim, to test the application of social norms theory to prevent the sexual exploitation of children and adolescents, the LINEA Intervention’s primary modifiable determinants are social norms.

### 3.3. Stage 3: defining mechanisms of change

During a 2017 inception meeting we collaboratively developed a theory of change (ToC) for the LINEA Intervention. The inception meeting included representatives from the UK and Tanzanian research institutions that carried out the formative research, the Tanzanian implementing partner organization AGH, the radio drama production organization MFDI, curriculum development experts, and funders.

The LINEA Intervention ToC was informed by the LINEA Intervention formative research, social norms theories, and the expertise of professionals working in the field of preventing violence against women and girls and prevention of sexual exploitation of children in Tanzania. The LINEA Intervention ToC included six phases, which are flexible, mutually reinforcing, non-linear, and can take place at any stage in the intervention (Table 3). The ToC also incorporates two central pathways to change. The first was to strengthen individuals’ knowledge, attitudes, and skills to support adolescent girls’ development, reflected in phases 1–3 of the LINEA Intervention ToC. The second directly operationalizes social norms theory to transform the social norms and unequal power dynamics that uphold ADTS. This is reflected in phases 4–6 of the ToC. The LINEA Intervention components and activities facilitate these two central pathways to create new social norms which are protective against ADTS. A structural component would constitute a third pathway to change: improving structural and material conditions to healthy developmental outcomes for adolescent girls. To account for this the intervention was designed to act alone or to accompany structural interventions as a ‘plus’ component.

**Table 3:** Learning Initiative on Norms, Exploitation, and Abuse (LINEA) Intervention theory of change.

1. Reflect on values (and highlight how they align or differ from existing norms and behaviors)	Participants critically reflect on whether their values align with adherence to harmful social norms, to provide a motivation for participants to change.
2. Build knowledge and skills	Participants gain knowledge and guidance about recognizing, avoiding, and preventing ADTS. Participants practice what they have learnt outside of intervention activities, with support from their peers and intervention staff.
3. Synthesize values with new knowledge and skills	Participants align personal and group values with new knowledge and skills. Participants gain motivation to learn and act reinforcing phase 2.
4. Shift to protective social norms	Participants adopt aspirational new norms, which are protective against ADTS. Examples include the expectation that fathers should actively support their daughter to avoid ADTS, or the expectation that adults should support adolescent girls to say ‘no’ to ADTS.
5. Support each other in norm change	New reference groups adhering to protective norms are formed among intervention participants and their wider communities through diffusion. Intervention participants adopt bystander behaviors to question tolerance of harmful norms.
6. Make commitments and act	Intervention participants make commitments to each other and intervention staff to jointly adopt new norms and collectively resist backlash and sanctions for non-adherence to norms that drive ADTS. This stage reinforces phase 5.

### 3.4. Stage 4: clarifying delivery of change mechanisms

The LINEA Intervention change mechanisms and two-component intervention proof of concept comprised a compressed radio drama and a curriculum targeting adolescent girls and adult couples with an adolescent daughter. The curriculum was based on the theory of change (ToC) and drew on emerging evidence about the success of couples-based interventions to prevent gender-based violence (Abramsky et al., 2014; Stern et al., 2018; Clark et al., 2020).

In comparison with the LINEA Intervention development process the 6SQuID framework does not provide detailed guidance on establishing delivery of change mechanisms. For the LINEA Intervention the change mechanisms were determined through feasibility testing (restricted to the Stage 5 in the 6SQuID framework). Based on the feasibility study feedback from radio drama listener groups the radio drama proof of concept proved relevant and acceptable with no evidence of harmful unintended consequences. In contrast, we made significant changes to the curriculum component through consultation with curricula development experts. One unexpected change was the decision not to work with adult couples, as is common in interventions to prevent intimate partner violence. Instead, we developed two curricula to maximize relevance and appropriateness for the curriculum participants: groups of adolescent girls (13–15), and adult men. Based on observed linkages between the intervention components curricula case studies were synchronized with radio drama characters and storylines. Table 4 describes the refinements to maximize the acceptability and relevance following feasibility testing.

**Table 4:** Comparison between the learning initiative on norms, exploitation, and abuse (LINEA) intervention proof of concept, and the finalized LINEA intervention.

	Modality of delivery		Intervention participants	
	Proof of concept	Finalized LINEA intervention	Proof of concept	Finalized LINEA intervention
Radio drama	15 Radio drama scenes	• 39 20-min radio drama episodes in three 13-episode seasons • Serialised on a local radio station, or audio files and listening devices distributed to listener discussion groups	Whole community	Whole community, listener discussion groups
Curriculum	12 Two-hour curriculum sessions	• 17 90–120-min sessions for adolescent girls (aged 13–15)• 18 120-min sessions for adult men	• Adolescent girls (aged 13–15)• Adult couples with an adolescent daughter	• Adolescent girls in school (aged 13–15)• Adult men (25 and over) working on male dominated activities

This LINEA Intervention development phase went well beyond what was proposed in the 6SQuID framework. Nonetheless, LINEA’s process corresponds with Stage 4 of the 6SQuID framework in several ways. First, we clarified the conditions and resources for successful delivery of the two LINEA Intervention components through feasibility testing. Second, as proposed in the 6SQuID framework, feasibility testing also enabled us to identify and mitigate harmful unintended consequences. For example, during curriculum session observations, we noted that generic content on consent and coercion in ADTS risked reinforcing victim-blaming attitudes if not designed specifically for adolescent girls. Third, we worked with local implementers to deliver the intervention and incorporated their feedback into the feasibility study results.

### 3.5. Stage 5: testing and adapting the intervention

During multiple phases of feasibility testing and refinement, we explored the processes and indications of change from the LINEA Intervention. The 2018 proof of concept feasibility study had three central objectives: (1) assess the intervention’s community acceptance, and contextual and cultural relevance; (2) understand any programmatic challenges and opportunities for the successful delivery of the intervention; and (3) identify indications of the intervention’s impact, as a full experimental study was not possible at this stage. We carried out a separate, incremental process to finalize the radio drama, described in the case study below.

The evidence on feasibility testing aligns with the 6SQuID framework’s recommendation to collect data to inform the intervention delivery, content, relevance, and acceptability. However, in the 6SQuID framework, feasibility testing is restricted to Stage 5, whereas for the LINEA Intervention it was ongoing and part of an incremental testing and revision process. While Wight et al. (2016) suggest that feasibility testing is commonly the most hurried stage of intervention development, substantial time and engagement was invested in the LINEA Intervention’s testing and refinement. The LINEA Intervention also diverged from the 6SQuID guidance as we did not carry out an economic costing or evaluability assessment with an experimental design at this stage.

#### 3.5.1. Case study: iterative radio drama development process

**Figure 2:**
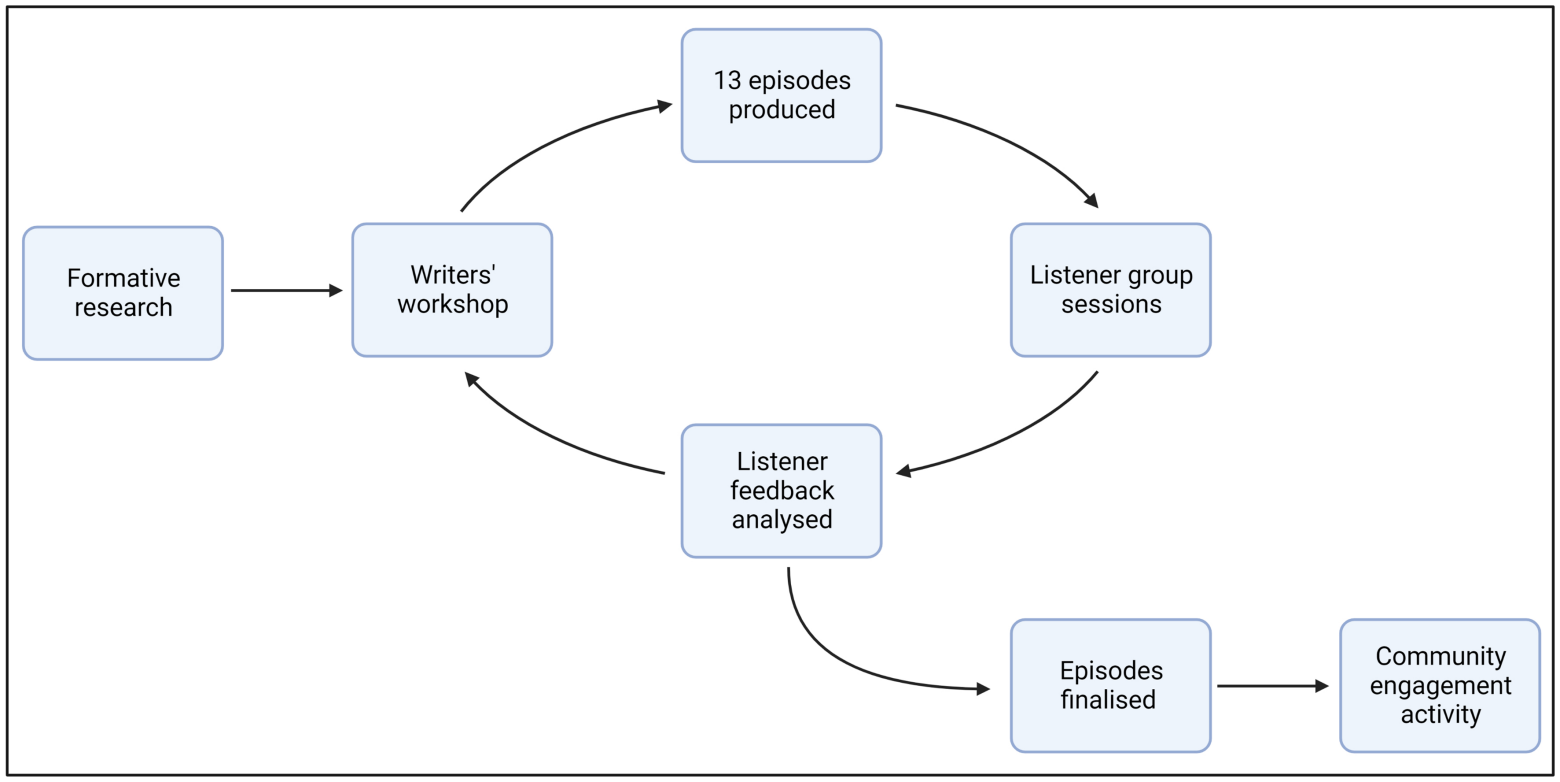
Iterative radio drama development process.

The 39-episode radio drama was developed iteratively in 2019 over three waves of feedback from listeners (Figure 2). In an initial workshop LINEA researchers, AGH, and MFDI developed the characters and a storyboard for all episodes from the formative findings. MFDI developed Act 1 (13 episodes), and AGH collected feedback in listener group sessions with 16 adolescent girls (aged 13–15), adult women, adult men, and community leaders, which was analyzed by the LINEA team. Team discussions then informed storyboards for the next act. This process was repeated until all 39 episodes of the radio drama were completed. MFDI developed a guide for broadcasters and a discussion guide to help with future delivery of the radio drama. LINEA, AGH, and MFDI jointly held a community engagement activity with research participants to conclude the process and foster a sense of ownership of the radio drama for the implementing partners and community members who participated.

### 3.6. Stage 6: collecting evidence of effectiveness

We assessed the feasibility and effectiveness (using indications of change) of the proof of concept for the radio drama and curriculum components separately. Participants found the radio drama proof of concept relevant and engaging, and the casting and ambient sounds realistic. The curriculum proof of concept for girls and adult couples was broadly acceptable and contextually and culturally relevant. Attendance was high in all participant groups. Evidence suggested that participants started to reflect critically on their own experiences of ADTS. One adult male participant stated:

*“There was a session where I felt like they were talking about me. The session about transactional sex. […] It made me feel like I should change.” (Adult man curriculum participant, post-participation interview)*

As recommended in the 6SQuID framework, we tested for evidence of harmful unintended consequences and found none for either component. Further testing of the radio drama component occurred in two studies in the Kigoma and Shinyanga regions of Tanzania in 2021. Forthcoming results explore monitoring of potential harm, or backlash; dose–response testing of modalities of delivery (household-level radio drama discussion groups versus community-level broadcasts of the radio drama); and transferability to different geographical contexts and populations (households including one or more person with a disability). All findings from the LINEA Intervention process suggest that the intervention is ready to be implemented at a larger scale. If the results of the upcoming pilot randomized controlled trial shows promise, findings will inform a larger scale trial of scaled-up or adapted versions implemented in other areas of Tanzania.

## 4. Discussion

In this paper, we reflected on the LINEA Intervention development process by retrospectively mapping it to the 6SQuID framework. We chose the 6SQuID framework because it provides pragmatic guidance for a phased approach to intervention development. Our comparison found three important differences between LINEA and the 6SQuID framework: (1) non-linearity of intervention development; (2) multiple stages of feasibility testing and refinement; and (3) collaboration between research and locally based implementing partners throughout the intervention development process.

We found that the LINEA Intervention development process and the 6SQuiD framework aligned in many respects. Each of the six stages of the 6SQuID framework were present in the LINEA Intervention development process and broadly occurred in the same sequence. To design the LINEA Intervention we invested significant time and resources in formative research and iterative feasibility testing and refining, which align with 6SQuID Stages 1, 4, and 5. The contextual factors and change mechanisms identified in the LINEA Intervention development process reflected an expressed aim of testing social norms change theories, and so had a narrower focus than suggested in Stages 2 and 3 of the 6SQuID framework. Finally, our collaborative approach to intervention development meant we assessed the appropriateness of further pilot testing (6SQuID Stage 6) by considering indications of change and monitoring unintended consequences gathered through routinely collected data from intervention participants and partner staff delivering intervention components.

The LINEA Intervention development process invested significant time and resources in phases that Wight et al. (2016) describe as important but often overlooked: formative research, feasibility testing and refining. The LINEA Intervention incorporated elements of a clearly articulated behavior change theory, social norms theory, in multiple stages of the development process, which Wight et al. (2016) state is rare.

Importantly, our experience showed that the six stages of the process overlap and the LINEA Intervention returned to certain stages multiple times, whereas the linear stages of the 6SQuID framework have clear cut-offs between them (Wight et al., 2016). Our findings align with recommendations from the broader literature, which suggest that intervention development should be a dynamic, iterative process that is open to change, and forward-looking to future evaluations (Hoddinott, 2015; Bleijenberg et al., 2018; O’Cathain et al., 2019; Turner et al., 2019; Zimmerman et al., 2021). The LINEA Intervention radio drama development case study showed how certain intervention elements may benefit from intensive, cyclical, and iterative development. Examples from the LINEA Intervention development provide insight into how to incorporate a non-linear and iterative approach in the latter stages of the 6SQuID framework (see case study and Figure 2).

Feasibility testing was a central feature that occurred throughout the LINEA Intervention development process, which was intended to determine relevance and acceptability, minimize unintended consequences, and ultimately improve efficacy. In comparison, feasibility testing only occurs in Stage 4 of the 6SQuID framework. The 6SQuID framework also provides relatively little information about best practice for feasibility testing. This reflects a broader lack of guidance and published qualitative feasibility test data in public health intervention research.

The final difference relates to research collaborations. LINEA Intervention development has benefitted from long-standing collaborations between academic researchers in Tanzania and the UK, curriculum development and mass media experts, and implementing organizations with expertise in preventing violence against women and girls, and sexual exploitation. Many activities and processes central to this collaboration were missing from the 6SQuID framework (for example, LINEA’s extensive partner mapping and identification process).

**Figure 3:**
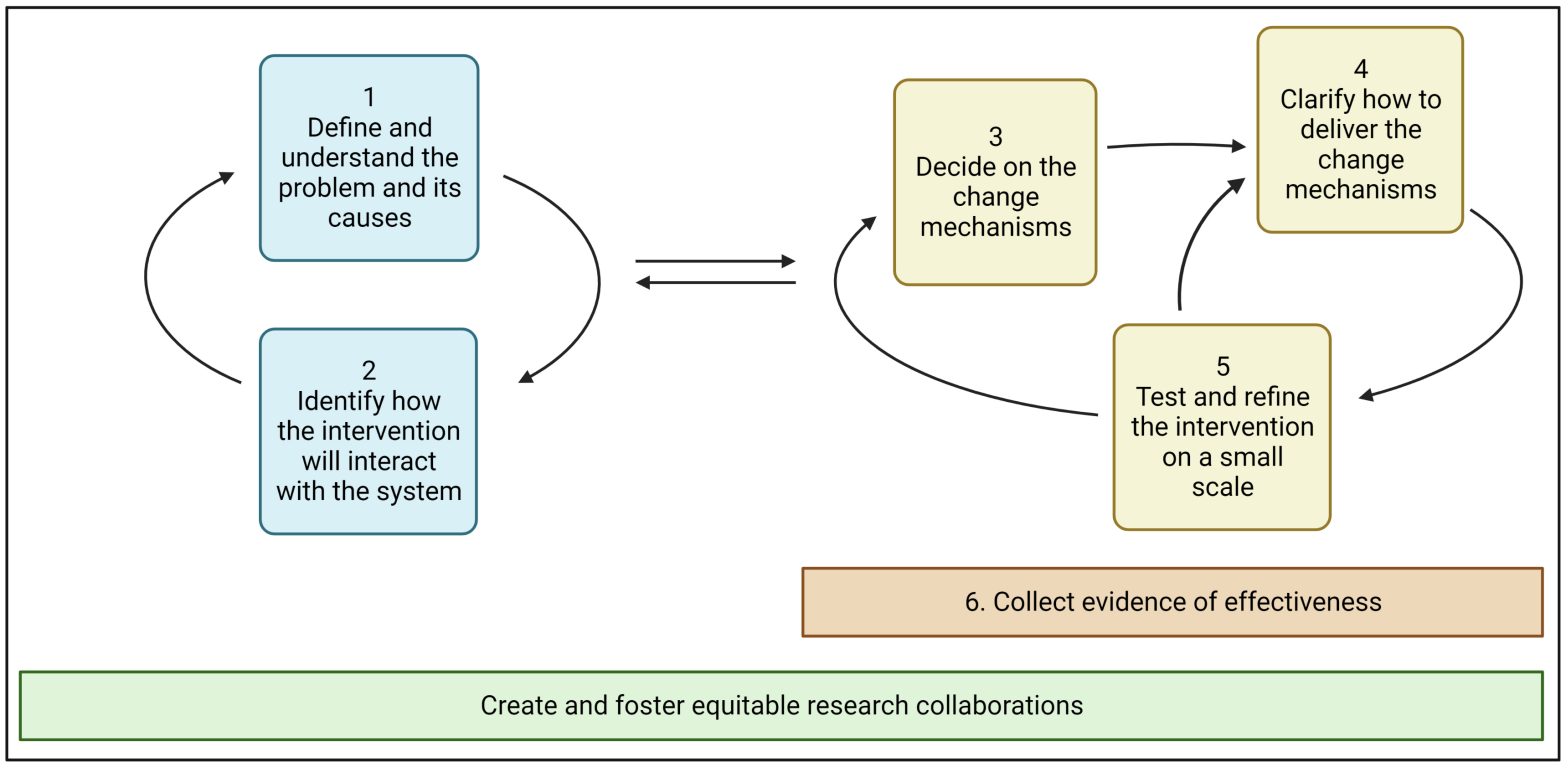
A dynamic and non-linear modification to the six essential steps for quality intervention development (6SQuiD) Framework.

Drawing on this learning we can suggest a modification to the 6SQuID framework (Figure 3). The six stages have remained the same but are no longer presented linearly. The modified framework suggests increased exchange and interaction between Stages 1 and 2, which concern problem identification. Stages 3, 4 and 5 are presented cyclically representing the need to iteratively test and refine the change mechanisms throughout the intervention development process. The modified framework also acknowledges that in the refining the change mechanisms and their delivery, intervention developers can also further define and understand the problem and its interaction with the system.

We propose that Stage 6—collecting evidence of effectiveness—can occur throughout the testing and refinement of the intervention change mechanisms. Collecting evidence in an on-going way allows a more flexible approach to assessing impact, creating opportunities to respond to adverse outcomes, and recognize possible positive unexpected outcomes. For example, further testing of the LINEA intervention in 2021 in preparation for a full pilot evaluation has found unexpected positive changes in caregivers’ feelings of responsibility to discuss ADTS with their daughters to prevent them from harm (Pichon et al., 2022). This change occurred despite the decision not to target adult couples who were caregivers of adolescent girls in the intervention design, as part of Stage 4 when we were clarifying the delivery of change mechanisms.

We have added an additional Stage called ‘Create and foster equitable research collaborations’ (shown in green in Figure 3), which cuts across all activities and should begin before Stage 1 starts. This Stage includes specific activities such as partner mapping and identification, but also influences how activities are conducted in the other six steps such as engaging implementing partners in testing and refining the intervention on a small scale.

There are some limitations to the intervention development research presented in this paper. Retrospectively applying the framework prevented the authors from rigorously and purposefully testing the framework. Instead, we compared it to a process that occurred organically, informed by emerging learning about intervention development from the wider field. The LINEA Intervention was developed over several years, with significant financial investment. It provides useful evidence for best practice given the current funding environment, which focuses on achieving outcomes rather than taking a longer-term view by investing in intervention development to optimize potential for success and minimize spending on ineffective interventions. This paper informs how we measure success for intervention development and will contribute towards more investment and thoughtful consideration of intervention development research.

### 4.1. Implications for research and practice

Despite these limitations our results offer lessons for future research and practice. Our research highlights the enormous benefits of formative research, feasibility testing, and refinement interventions, which are commonly given short shrift in intervention development and funding. The exercise of retrospectively applying the 6SQuID framework has underlined the need for intervention developers to review the literature on approaches to intervention development during the project inception phase. Intervention developers can turn to alternative research to guide feasibility testing (Eldridge et al., 2016; Ogilvie et al., 2020), which might help answer questions, such as how to establish if an intervention is ‘feasible enough’ and ready for piloting, given that intervention feasibility studies typically include small sample sizes and do not use experimental evaluation designs such as randomized controlled trials (RCTs).

A central lesson from our experience was the fundamental importance of collaboration with locally based implementing partners. There is a growing body of literature on the benefits of co-production and collaboration in research approaches (Voorberg et al., 2015; Hawkins et al., 2017; O’Cathain et al., 2019; Oliver et al., 2019; Williams et al., 2020). Collaborative research can create a mutually beneficial exchange for all parties and recognizes the need to address challenges that arise in such relationships by incorporating flexibility, investment of time and resources, and open communication (Zimmerman et al., 2016; Oliver et al., 2019). Collaborative approaches to intervention development require significant investments of time and resources, and sensitive management of the priorities and relative decision-making power of different actors (Oliver et al., 2019), in this case, academic and NGO partners, plus other stakeholders including funders.

## 5. Conclusion

This paper contributes evidence to encourage the importance of intervention development methods and adopting a flexible and iterative approach. In our theory-driven intervention, this meant developing a locally relevant theory of change and change mechanisms to address patriarchal inequalities. Ultimately, we find that the 6SQuID framework offers useful guidance for developing a locally informed intervention. However, we suggest that future intervention development research should incorporate greater use of co-production and iterative phases of design and adaptation to take account of the non-linear nature of behavior change. Our research provides an important alternative vision from short-sighted funding to longer-term investments in the development phase of an intervention, to optimize the potential to create efficient and effective interventions to prevent gender-based violence.

## Data availability statement

The datasets presented in this article are not readily available because the data are qualitative and not possible to anonymise. Requests to access the datasets should be directed to AMB, ana.buller@lshtm.ac.uk.

## Ethics statement

The studies involving human participants were reviewed and approved by London School of Hygiene and Tropical Medicine, and the National Institute of Medical Research, Tanzania. Written informed consent to participate in this study was provided by the participants’ legal guardian/next of kin.

## Author contributions

LH-M drafted the manuscript, conducted data analysis, and contributed to data collection and intervention development. AMB conceived of the study, drafted the proposal, obtained funding, and provided oversight for all elements of data collection, analysis, intervention development and manuscript production. CZ contributed to conceptualization and production of the manuscript. RS, JR, PP, LR, and RY contributed to intervention development. RS also oversaw elements of data collection. JW oversaw study design, data collection, and analysis for the LINEA Intervention formative research. All authors contributed to the article and approved the submitted version.

## Funding

This research was funded by the OAK Foundation (Grant No. OCAY-16-180) and Wellspring Foundation (Grant No.13343).

## Conflict of interest

Author LR was employed by Lori Rolleri Consulting Inc.

The remaining authors declare that the research was conducted in the absence of any commercial or financial relationships that could be construed as a potential conflict of interest.

The reviewer KD declared a past co-authorship with the author JW to the handling editor.

## Publisher’s note

All claims expressed in this article are solely those of the authors and do not necessarily represent those of their affiliated organizations, or those of the publisher, the editors and the reviewers. Any product that may be evaluated in this article, or claim that may be made by its manufacturer, is not guaranteed or endorsed by the publisher.
